# Delphi consensus on add-ons and social midia in Assisted Reproductive Technology

**DOI:** 10.5935/1518-0557.20230047

**Published:** 2023

**Authors:** Alvaro Ceschin, Álvaro Petracco, Edson Borges Jr, Emerson Barchi Cordts, Hitomi Miura Nakagawa, Maria do Carmo Borges de Souza, Maria Madalena Pessoa Caldas, Newton Eduardo Busso, Paulo Gallo de Sá, Pedro Augusto Araújo Monteleone, Rui Alberto Ferriani

**Affiliations:** 1 Feliccità - Instituto de fertilidade, Curitiba, PR, Brazil; 2 Fertilitat - Porto Alegre, RS, Brazil; 3 Fertility Medical Group, São Paulo, SP, Brazil; 4 Associação Instituto Sapientiae, São Paulo, SP, Brazil; 5 Instituto Ideia Fértil, São Paulo, SP, Brazil; 6 GENESIS - Centro de Assistência em Reprodução Humana, Brasília, DF, Brazil; 7 Fertipraxis Centro de Reprodução, Rio de Janeiro, RJ, Brazil; 8 Clínica de Fertilidade GEARE, Recife, PE, Brazil; 9 Projeto ALFA, São Paulo, SP, Brazil; 10 Centro de Fertilidade Vida, Rio de Janeiro, RJ, Brazil; 11 Monteleone - Centro de Fertilização Humana, São Paulo, SP, Brazil; 12 Setor de Reprodução Humana FMRP/USP, Ribeirão Preto, SP, Brazil

**Keywords:** Add-ons, Assisted Reproductive technology, delphi consensus, social media

## Abstract

This article reports the annals of a national consensus meeting on add-ons and social networks in Assisted Reproduction Techniques (ART). The panel of experts has developed a set of consensus points and this document is intended to be referenced as a national consensus to allow social networks and add-ons to be used in ART, following the standards of the Code of Medical Ethics and the Federal Council of Medicine, in a safe ethical and responsible way.

## INTRODUCTION

Data from the report by SisEmbrio - National Embryo Production System, published by the National Health Surveillance Agency (ANVISA), show the Brazilian role in relation to the use of Assisted Reproduction Techniques (ART) (ANVISA, 2022). Concomitantly, certain cultures have developed in digital spaces that promote connectivity, open sharing and self-disclosure (Bazarova & Choi, 2014; Moorhead *et al*., 2013). In the last decade, digital professionalism has been defined as “the attitudes and behaviors reflecting traditional paradigms of professionalism that are manifested through digital media” (Cain & Romanelli, 2009).

In medicine, social media offers numerous opportunities including networking and collaborating with colleagues, receiving and giving advice, learning new knowledge and skills (Cheston *et al*., 2013; O’Connor *et al*., 2018; Sterling *et al*., 2017), sharing useful health information with patients and the public (Gholami-Kordkheili *et al*., 2013), lobbying in government to change policy or inform health policy (O’Connor, 2017). However, students and health professionals use social media and interact with others in virtual environments in a non-professional way (Kaczmarczyk *et al*., 2013). In the United States, executive directors of medical boards reported that the most common violations of online professional behavior included inappropriate communication with patients, misrepresentation of qualifications, or use of the Internet for inappropriate practices, such as prescribing medication (Greysen *et al*., 2012). In some countries, disciplinary action has been taken by regulatory bodies due to unprofessional behavior and values displayed by healthcare professionals online (Mansfield *et al*., 2011; Rimmer, 2017).

There are reports that healthcare students’ and physicians’ perceptions of digital professionalism on social media are mixed (Neville & Waylen, 2015; Gettig *et al*., 2016; Hall *et al*., 2013; Chretien *et al*., 2010). Lefebvre *et al*. (2016) suggest that there is a link between being educated about digital professionalism and exhibiting more cautious behaviors online. Indeed, some healthcare regulatory associations have produced guidelines on the responsible use of social media (Australian Health Practitioner Regulation Agency, 2020; National Council of State Boards of Nursing, 2020). However, there are still no guidelines for the use of digital professionalism in ARTs to be done responsibly.

Another ethical dilemma that has been much discussed nowadays are the adjunct interventions - called add-ons - that have been introduced into the routine of some assisted reproduction centers. Add-ons have been defined by the Human Fertilization and Embryo Authority (HFEA) as “additional optional treatments” that are not essential to ART but may be offered alongside “standard” treatments, usually at an additional cost (HFEA, 2019a). They include a collection of old and emerging technologies; however, they are unregulated and supported by limited or no evidence.

In view of the observed aspects, our objective is to develop a consensus on the responsible use of social media and add-ons regarding ART. Among the qualitative research methodologies, the Delphi method is a powerful investigation technique (Facione, 1990), as it allows gathering a set of geographically separated specialist opinions, leading to dense results on complex and comprehensive themes. Such potential makes it possible to make profound readings of reality and serves as a basis for a better understanding of the phenomena and, mainly, also to guide informed decision-making and transform reality based on the opinions of the intervenient and the specialists involved.

## METHODS

The Delphi method is defined as “a method for structuring a collective communication process so that it is effective in allowing a group of individuals, as a whole, to deal with a complex problem” (Linstone & Turoff, 2002). It consists of a set of questionnaires that are answered, sequentially, individually by the participants, with summarized information about the group’s responses to the previous questionnaires (Osborne *et al*., 2003), in order to establish a kind of dialogue between the participants and, gradually, build a collective response. The results are analyzed by the researchers between each round of questionnaires. Tendencies and dissonant opinions are observed, as well as their justifications, systematizing and compiling them to later resend them to the group. Thus, after knowing the opinions of the other members and the group’s response, the participants have the opportunity to refine, change or defend their answers and send them back to the researchers, so that they can redesign the new questionnaire based on this new information. This process is repeated until a consensus is reached (Grisham, 2009). There is a core of common characteristics that define and distinguish this technique from others. They are: i) anonymity; ii) feedback on individual contributions; iii) construction and presentation of the group’s response as a whole; iv) possibility of reviewing and changing responses (Linstone & Turoff, 2002; Osborne *et al*., 2003). The process of implementing the Delphi method takes place in several steps, which, according to the consulted literature (Linstone & Turoff, 2002; Grisham, 2009), can be divided into:

1. Choice of Expert Group.
2. Construction of the questionnaire 1.
3. First contact with experts and invitation to participate in the research.
4. Sending the questionnaire 1.
5. Receipt of questionnaire responses 1.
6. Qualitative and quantitative analysis of responses.
7. Construction and sending of questionnaire 2 with feedback.
8. Receipt of responses to questionnaire 2 and their analysis.
9. Sending the following rounds of questionnaires, interspersed with the respective analyses.
10. End of the process and writing of the final report.

**Figure 1:**
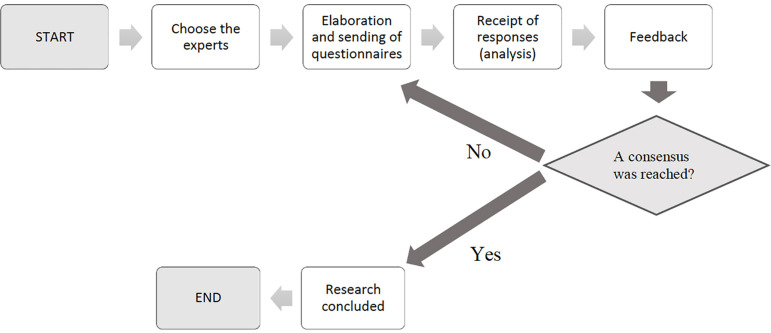
Generic implementation scheme of the Delphi method.

Each of these steps must be carefully prepared and implemented, and the entire process must be recorded and described. Next, we describe the main characteristics and factors to be considered in the referred steps. A scheme of the method implementation can be seen in Figure 1.

For this consensus, 11 experts were invited to answer a questionnaire containing 15 questions using the Google forms tool. Two questionnaires were necessary so that the pre-established level of agreement for consensus (≥ 70%) was reached for all questions. For 14 questions, participants rated their agreement with the statements using an agree, partially agree, and disagree scale. For question 6, a different scale was used, as can be seen below.

### Questions

#### Social media

1. Aware that science needs funding to be developed, I believe that the commercial interest of institutions can interfere with the results of published works, which can lead to the indiscriminate use of diagnostic and therapeutic tools in clinical practice.

2. I believe that the commercial interest of an institution can interfere with the results of sponsored studies, and if the physician is not aware, he can be influenced consciously or not.

3. I believe that Assisted Human Reproduction should be treated as a means activity, offering investigation and treatment of infertility, and not as an end activity, promoting the gestation and birth of a healthy child, since these outcomes cannot be guaranteed.

4. I do not change my behavior on social networks through the behavior of my competitors, but when needing to change my strategy, I remain ethical and faithful to my principles and convictions.

5. It is correct to hire specialists in social media in order to publicize the activities of the medical professional who works in Assisted Reproduction, provided that the texts are guided and reviewed by the medical professional, taking into account the limitations and prohibitions in marketing practices in the medicine proposed by CFM - *Conselho Federal de Medicina* - together with CODAME, Commission for the Disclosure of Medical Affairs.

#### Add-ons

6. In the presence of two divergent positions on the same subject in the literature and in the decision to adopt this treatment, how would you choose between one and the other? (Response options “based on my experience”, “according to the patient’s decision”, and “would contraindicate the treatment”).

7. Due to various failures in IVF/ICSI, add-on treatments, despite not having robust scientific evidence, can be offered, depending on the case in question, the add-on and the full explanation to the patient about the lack of scientific evidence.

8. The specialist physician has a greater contribution to the decision to use add-ons than the patient herself - he must establish an acceptable limit for the use of a certain technology, not adopt experimental procedures with proven harm, and must be the one who determines the conduct and not the patient.

9. The exposure of add-ons on social media can be considered ethical despite the lack of scientific proof or consensus on their effectiveness, provided that the level of scientific evidence is clarified, the fact that they may be beneficial only in some specific cases, and the increase in the value of the treatment.

10. We can use add-ons, if that is the patient’s will, regardless of scientific evidence, as long as the risk/benefit and cost of the add-on are well evaluated, remembering that the medical act is a medical responsibility and should not be left surrendering to the lay requirement, injuring medical autonomy and good clinical practice, and it is up to the physician to demolish idealizations that do not benefit the case, justifying the points involved.

11. The couple influenced by the exposure of add-ons in the media, and their decision to use them, authorizes the doctor to offer these treatments, if they are discussed with the patient, do not bring proven harm, and consent to alternatives is requested devoid of robust scientific evidence. The doctor must be responsible for clarifying why the alternatives exposed in the media would not benefit the case and could even cause physical, emotional, financial harm, etc. It is up to the doctor to explain the scientific evidence on the subject or the proposed technique and say whether he agrees with its use. The doctor must be sovereign in his conduct and must not be induced to use a technique that he does not agree to use.

12. Physicians are driven to propose add-ons, most of the time, out of a genuine desire to increase pregnancy rates, especially in cases of recurrent implantation failure. Other motivations include patient retention, self-promotion, and extra gain, associated with distortion of medical practice.

13. The prescription of add-ons, based on the genuine desire to increase pregnancy rates, after discussing their advantages and disadvantages with the patient, can be considered ethically acceptable - provided that the add-on in question does not cause proven harm, and the treatment is well documented and consented by the patient.

14. Taking into account all aspects (positive and negative), social networks are useful for patients (or couples) as a “call for debate” on add-on techniques, leaving it up to the specialist doctor to discuss with the patient about the treatment in question based on reliable sources of information such as FEBRASGO, SPMR, SBRA, SBRH etc. However, there are still no adequate filters to moderate the information propagated by social networks, which can result in anxiety and other negative feelings in the patient.

15. In order to offer a treatment to my patients, it must have been previously proven by well-established scientific studies. However, in handling exceptional situations, a treatment without scientific proof due to lack of data or robust studies, or any other reasons, can be used, with the patient’s consent and proper documentation.

## Results

**Figure:**
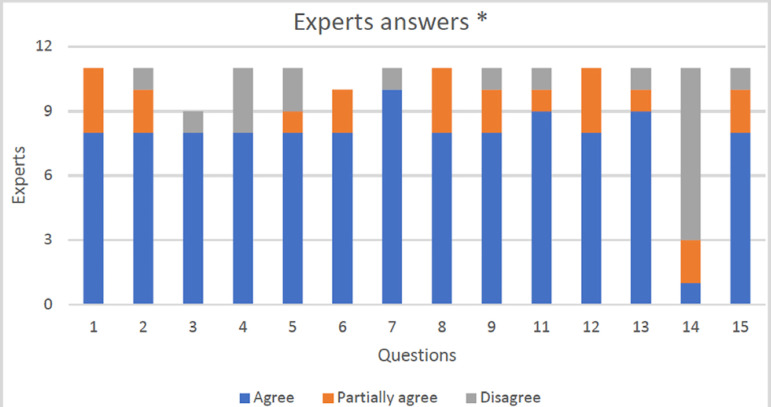
* Question 10 was answered differently from the rest, in which ten experts answered “based on my experience”, and one specialist answered “he would contraindicate the treatment”.

## DISCUSSION

### Social media

The doctor-patient relationship was regulated by the Nuremberg Code (The Nuremberg Code, 1949) after the Second World War, due to the barbarities committed until then, mainly by Nazi doctors. In 1964, the 18^th^ General Assembly of the World Medical Association approved the Declaration of Helsinki (World Medical Association, 2001), which established ethical principles for health research involving human beings, which also had an impact on the doctor-patient relationship. Currently, the internet tool that came with the development of technology, ended up benefiting the doctor-patient relationship, fostering discussions about the therapeutic conduct, due to the large amount of medical information that it makes available. However, the lack of parameters of conduct and effective specific regulation in Brazil can compromise the confidentiality and privacy between doctor and patient, and propagate the dissemination of irresponsible information about health (Wechsler *et al*., 2003), and thus should be considered a potentially problematic public health issue (Moretti *et al*., 2012). Thus, this Delphi consensus aims to establish parameters for professional practice, so that respect for others prevails in accordance with Brazilian ethical and legislative requirements.

The technological revolution has changed the way of obtaining information, the forms of communication and even the interaction between people. Social media, in addition to facilitating information, is able to endow its users with a certain power over it, even as content producers, enabling the dissemination of the circulation of different moralities. The use of social media conveys values and standards of conduct, in addition to socializing generations by bringing people together. However, it comes with its onus and bonuses, dictating rules and changing behaviors, bringing both benefits and harms (Toral, 2006; Setton, 2002).

Social networks have more than 150 million users in Brazil (Monitor Mercantil, 2021). The internet is the population’s main source of information about health problems. It is undeniable that social networks have revolutionized communication between people and, therefore, are very effective in the medical marketing strategy, generating greater visibility, transmitting credibility, attracting, and retaining patients, thus strengthening the digital presence. However, medical advertising is regulated by a series of rules to guarantee the good practices of health professionals. One of them is the Code of Medical Ethics (CEM) updated in 2019 with the purpose of contemplating these changes brought about by the digital transformation and complemented by two resolutions (n.º 1974/11 and 2126/15). The norms established by CEM prohibit the dissemination of photos of patients, the use of terms that convey the idea that their service is superior to that of other professionals, disallow consultations or prescription of medication in channels aimed at mass communication or distance, requires the inclusion of personal data, enrollment numbers in the Regional Council of Medicine (CRM) and the Physician’s Specialist Qualification Register (RQE) in all advertising material or information from the technical director responsible for legal entities.

Resolution n.º 2,126/15 covers medical ethics on the internet and social networks and advises that specialties not recognized or registered in the Medical Councils cannot be announced or act in the media, prohibits what techniques or methods not recognized by the CFM are advertised, does not authorize publications that characterize sensationalism, self-promotion, or unfair competition, prohibits posts in the format “before/after” the performance of procedures. The observance of these restrictions, in addition to benefiting patients, values the physician’s ethical conduct and protects him from possible lawsuits filed by third parties.

Resolution CFM nº 1974/2011 establishes criteria that guide advertising in Medicine, determining the rights of professionals and conceptualizing advertisements, disclosure of medical matters, sensationalism, and self-promotion. Medical advertising is not comparable to the advertising of purely commercial products and practices and must exclusively obey ethical principles of educational guidance. The resolution also defines “medical publicity”, “announcement” or “propaganda”, as the communication to the public, by any means of dissemination, of professional activity of initiative, participation and/or consent of the physician. It is also prohibited for the doctor to share information that may imply promises of results in the treatments offered, because, if he does not obtain them, an indefensible situation is created.

This appears to be the norm most violated when medical data on social media is analyzed. It is also prohibited to disclose the address and business telephone numbers, clinic, or service by the doctor, when he acts as a propagator of information on social media. In short, the CFM regulates and guides physicians not to produce inappropriate advertising that could be accused in legal proceedings.

Thus, it is important to understand how social networks can impact the doctor-patient relationship and how to separate personal and professional life in virtual environments, differentiating the personal profile from the professional, confirming the source before sharing information in the various social networks currently available. On Tik Tok, the social network of the moment, users share short videos mainly of dances and voiceovers. Therefore, students and health professionals have also used this environment a lot to share information. It is very common to watch videos of disclosure and self-promotion of their activities, which violates CFM rules. However, it should be noted that informative videos with strictly educational purposes are allowed. YouTube is also often used for educational purposes. However, its academic application can be compromised by the lack of guarantee of veracity of the shares. The ease and speed of interaction between physicians and patients made possible by the WhatsApp phone application also generated a position by the CFM in 2017, favorable to the use of the application, but also limiting its use (Nazareth *et al*., 2020). Observing the norms of not diagnosing, not prescribing therapies, and not replacing the medical consultation with remote conversations, the use of the application for the purpose of obtaining medical information was considered healthy. However, once the contact is made available to the patient, omission or negligence may be characterized as the lack of response to a message sent by the application.

### Conflicts of interest in scientific publications

With the increasing importance of scientific research, the situation in which the results of research projects contradict or promote personal, professional, and institutional interests has become more frequent. Currently, it is much discussed among agencies that promote projects, universities, researchers, and companies how to measure the veracity of scientific publications. During the development of a research project, the interests of several participants are involved, such as the research sponsor, the researcher, and society itself, whether converging or diverging, which can configure the “potential conflict of interests”. In the absence of parameters for measuring decisions related to conducting the research or determining the data set, the quality of the decisions taken depends on the researcher’s “methodological good sense”, which can be involuntarily hurt by potential conflicts of interest. Given that the complete avoidance of conflict of interests can only be achieved if a researcher does not get involved in projects whose results may run counter to his personal interests, and a company or institution does not sponsor projects whose results may run counter to his economic or institutional interests, a situation that is almost unlikely, more complicated strategies have been suggested for dealing with potential conflicts of interest. A well-known example is the requirement to disclose research funding sources for publishing articles in most scientific journals. Also, the data and procedures used in carrying out the project must be available for verification by auditors, so that the results are subjected to critical treatment before being accepted as scientifically validated results.

### Theory of obligation of means and result

The theory of means and result obligations emerged after the Industrial Revolution. Through it, the type of obligation is defined between the parties from the time the service is contracted, aiming at preventing difficulties related to the eventual civil liability of professionals in the event of an error that results in harm to the patient. In general, the obligation of means applies to most medical services, including professionals in the ART area, that is, the doctor must use all methods with the greatest possible care, aiming to achieve the desired result, in the case of the pregnancy that results in a live birth, but is not required to deliver that result.

The consent form for assisted human reproduction techniques is a contract entered into between the patient and the assisted human reproduction center, bringing with it information regarding the procedure to be performed, addressing both the characteristics and possible benefits, complications, and risks, as required. of the Consumer Protection Code. In other words, the patient, being aware of what he is consenting to, accepts the provision of services to be provided by the assisted reproduction center. The term of consent for assisted human reproduction techniques contains an affirmative clause in the sense that the clinic assumes an obligation of means and not of result. However, it is worth remembering that the exchange of information during the reading of the consent form is very important so that the type of obligation promised can be correctly defined, and there should be no verbal promises by the health professional regarding the guarantee of pregnancy, birth, baby health etc. It should be noted that regarding medical behavior on social networks, the information provided by professionals must be very well analyzed and disseminated, as it can also be collected by the patient at an opportune time.

Therefore, even if the document states that the human reproduction center assumes an obligation of means, there is the possibility of modifying the nature of that obligation to an obligation of result, depending on the answers given to the questions raised and what is publicly propagated by the health professional or assisted human reproduction center.

### Biased medical behavior / Outsourcing of medical writing

Many professionals have joined social networks to keep up with the trend. It is not uncommon to see the propagation of confusing and even dubious content, without scientific evidence and with the potential to manipulate the reader or follower. In medicine, interest in regulating online professionalism has been increasing considerably and has been amplified by an unprecedented and relatively recent event, where a controversial article was retracted in the Journal of Vascular Surgery. The article addressed what the authors considered to be inappropriate content generated by surgeons and caused widespread mobilization among social media users who used the hashtag #MedBikini to protest against connotations of sexism and misogyny (Sosa, 2020).

The development of texts, posts and other digital content requires skill, time and disposition. For these reasons, it is not uncommon to hire professional writers to provide this service. However, it is important that outsourced medical content writing provides highly specialized quality content. The principle of outsourcing, called 3E (effectiveness, efficiency and economy) proposed by Lele (2016) outlines the main areas of consideration for outsourcing in the context of knowledge-based functions in healthcare. “Effectiveness” refers to the delivery of quality, regulatory-compliant, consistent and reliable content. “Efficiency” is defined as the ability to manage peak workloads without greatly impacting productivity and costs. “Savings” refers to reducing costs without compromising quality and compliance.

A series of considerations must be weighed by health professionals when maintaining an online presence. In addition to being aware of patient privacy and confidentiality standards, physicians must continually monitor their online posture to ensure appropriate professional boundaries. For this, it is interesting to separate professional and personal profiles. In the case of outsourcing medical writing, it is extremely important for the physician to support the design of the content to be posted, as well as its revision before publication. It is still up to the physician, when apparently viewing non-professional content posted by professional colleagues, to bring this content to the individual’s attention, so that appropriate measures can be taken. Violation of professional standards must be reported to the competent authorities.

## CONCLUSION

Social networks have revolutionized the way information is currently sought. However, depending on how it is used, it can bring both benefits and harm. Medical advertising is regulated by different bodies and through a series of norms that health professionals must be aware of, so that they can remain continuously vigilant regarding their behavior during the online presence. Health professionals should be aware that this behavior can compromise their reputation both among patients and their professional colleagues, and thus have negative consequences for their careers and undermine the public’s trust in the medical profession. This Delphi consensus gathers information that guides the use of social networks by health professionals, advocating respect for others in accordance with Brazilian ethical and legislative requirements.

### Add-ons

Since the introduction of IVF techniques, fertility centers have continually sought to increase success rates. As a result, a growing variety of clinical adjuvant treatments, known as add-ons, have been used indiscriminately, thus generating a discussion about their efficacy and safety. Add-ons are optional extra, or adjuvant treatments often offered by ART specialists or even suggested by the couple or patient themselves, which involve an additional cost to the in vitro fertilization procedure, and which do not have reliable scientific evidence of benefiting the rate of live births. Currently, it is not uncommon to see a range of add-on options listed in a menu that is available for patients to choose the complements of their treatments. A recent study showed that at least one add-on was used in 82% of IVF treatments performed on 1,590 patients in Australia and 72% of them were charged an additional cost to the patient. Also, 83% of patients reported regret using add-ons after unsuccessful treatments, and 75% regretted it when the specialist had greater input in the decision to use add-ons (Lensen *et al*., 2021a). Before we specifically address the points highlighted by experts during the making of this consensus, we will discuss treatments currently referred to as add-ons below.

To facilitate the understanding of the scientific evidence for each add-on, we will use the traffic light classification, in the same way as the document issued by the Human Fertilization & Embryology Authority, which consists of three colors indicating evidence, in the form of randomized controlled trials (RCTs) of high quality proving the effectiveness of the add-on in increasing the chances of pregnancy, as follows:

● Green - add-ons with more than one high-quality RCT demonstrating that the procedure is effective in increasing the odds or shortening the time to pregnancy in some group of patients. These treatment adjuncts are routinely used in fertility treatments such as the use of intracytoplasmic sperm injection (ICSI) in couples whose cause of infertility is semen related.

● Yellow - add-ons that have contradictory evidence in RCTs demonstrating their effectiveness in increasing the odds or decreasing the time to pregnancy in some group of patients. In this case, since the evidence is not conclusive, more studies are needed for the add-on to be recommended for routine use.

● Red - add-ons that do not have evidence in RCTs demonstrating their effectiveness in increasing the chances or decreasing the time to pregnancy in some group of patients.

### List of add-ons (summarized in Table 1)

#### *Artificial oocyte activation with calcium ionophore* (Murugesu *et al*., 2017)

The physiological agent of oocyte activation was identified as a phospholipase Cz (PLCz) transmitted by sperm. During fertilization, entry of PLCz into the oocyte cytoplasm induces the release of calcium from the endoplasmic reticulum. The lack of calcium compromises the process of fertilization and embryonic cleavage. This disadvantage can be compensated for by artificially increasing the calcium in the oocyte and thus inducing its activation. Most commonly, artificial oocyte activation is induced by a variety of chemical agents or calcium ionophores, such as ionomycin and calcymycin (A23187).

**Table 1:** List of add-ons categorized according to traffic light scoring.

Traffic light scoring	Add-on
●	Artificial oocyte activation with calcium ionophore
	Elective embryo freezing cycles
●	Hyaluronate-enriched medium (eg, EmbryoGlue)
	DuoStim
	Intracytoplasmic Injection of Morphologically Selected Sperm
	Physiological Intracytoplasmic Sperm Injection
	Sperm DNA Fragmentation Test
	Preimplantation genetic testing for aneuploidy
	Timelapse imaging technology
	Growth hormones
	Male antioxidants
●	Assisted hatching
	Endometrial receptivity analysis
	Immunological tests
	Endometrial injury
	Endometrial microbiome metagenomic analysis
	Analysis of chronic infectious endometritis
	PAI-1 4G/5G polymorphism
	Seminal plasma and platelet-rich plasma
	Screening hysteroscopy
	Aspirin
	Heparin
	Intravenous immunoglobulin
	Glucocorticoids

Artificial oocyte activation with calcium ionophore is not recommended for most patients on ART, however, it can be effective in cases of low or complete failure of fertilization, or globozoospermia.

Traffic light classification: green ●

#### *Assisted hatching* (Lacey *et al*., 2021)

Failure of embryonic implantation can result from the inability of the blastocyst to escape its outer covering, known as the zona pellucida. The artificial opening of the zona pellucida is known as assisted hatching and has been proposed as a method to increase the success rate of assisted reproduction treatment, facilitating embryo implantation.

Traffic light classification: red ●

#### *Elective embryo freezing cycles* (Zaat *et al*., 2021)

Conventionally, IVF treatments consist of the transfer of a fresh embryo shortly after controlled ovarian stimulation, which may or may not be followed by one or more transfers of frozen embryos in subsequent cycles. Studies have suggested that the ovarian response to controlled stimulation may affect the uterine lining, making embryonic implantation difficult. Alternatively, one can choose to freeze all available embryos and transfer them after thawing in subsequent cycles, a strategy known as “freeze-all”, especially in cases with associated PGT, when there is a risk of developing OHSS, or when pituitary block with progesterone is used.

Traffic light classification: green ●

#### *Endometrial receptivity analysis (ERA)* (Zaat *et al*., 2021)

Endometrial receptivity is defined as intrauterine conditions favorable to embryonic implantation. Although the window of implantation (WOI) usually occurs over a 2- to 3-day period during the luteal phase, this period may be shifted or shortened in some patients undergoing IVF. ERA was developed in 2011 to analyze the endometrial molecular composition and try to identify the implantation window to decide the most appropriate moment for embryo transfer.

Traffic light classification: red ●

#### *Hyaluronate-enriched medium (eg, EmbryoGlue)* (Datta *et al*., 2015)

Culture media enriched with hyaluronic acid can be used during embryo transfer and supposedly favor implantation.

Traffic light classification: yellow ●

#### *Immunological tests* (Ali *et al*., 2018)

More than 50% of cases of implantation failure or recurrent pregnancy loss (RIF or RPL) are classified as idiopathic, that is, the factors are not identified. This category encompasses processes mediated by the immune response, such as the failure to establish maternal immune tolerance for implantation and acceptance of the embryo. Maternal imbalances in T helper lymphocytes and both peripheral and uterine natural killer cells have been suggested as possible factors. The role of testing these cells in patients with RIF and RPL is still controversial.

Traffic light classification: red ●

#### *DuoStim* (Vaiarelli *et al*., 2020)

DuoStim is an unconventional ovarian stimulation protocol, suggested for the treatment of cancer patients and poor responders, which combines two consecutive stimulations in the follicular and luteal phases of the same ovarian cycle, with the aim of maximizing the number of oocytes retrieved in a short period of time.

Traffic light classification: yellow ●

#### *Endometrial injury* (Lensen *et al*., 2021b)

In some cases, implantation of the embryo may not occur because the uterine lining (endometrium) is not favorable. Endometrial Injury, a procedure in which the endometrium is “injured” using a small sterile plastic tube, can be performed prior to Assisted Reproductive Technique (ART). The theory is that this procedure triggers the release of chemicals and hormones and the activation of genes that increase endometrial receptivity to the embryo.

Traffic light classification: red ●

#### *Intracytoplasmic Injection of Morphologically Selected Sperm (IMSI)* (Teixeira *et al*., 2020)

Morphologically selected sperm injection (IMSI) is the union of two techniques, MSOME (motile sperm organelle morphology examination) and ICSI, and allows the sperm to be observed at high magnification (> 6000x) thus favoring sperm selection for subsequent injection into the oocyte.

Traffic light classification: yellow ●

#### *Physiological Intracytoplasmic Sperm Injection (PICSI)* (McDowell *et al*., 2014)

Sperm sorting techniques supposedly increase the chance of selecting mature sperm with better DNA integrity for fertilization. The PICSI technique associates the selection of spermatozoa with the ability to bind to hyaluronic acid, with the ICSI technique, performed later.

Traffic light classification: yellow ●

#### *Sperm DNA Fragmentation Test* (Dai *et al*., 2022)

During the sperm cell maturation process, the DNA can be damaged, which may compromise the outcome of ART, especially the occurrence of spontaneous abortion. Several tests can be used to assess sperm DNA integrity. However, the evidence supporting the use of DNA fragmentation testing as a prognostic tool, to date, is conflicting.

In patients with a high rate of sperm DNA fragmentation, the application of advanced sperm sorting techniques such as PICSI and magnetism-activated cell sorting (MACS) resulted in higher clinical pregnancy rates than the use of gradient centrifugation. standard density (69.2%, 67.1% and 51.4%, respectively, *p*=0.025) (Hozyen *et al*., 2022).

Traffic light classification: yellow ●

#### *Preimplantation genetic testing for aneuploidy (PGT-A)* (Cornelisse *et al*., 2020)

One of the presumed causes of unsuccessful ART is that the embryos have an abnormal number of chromosomes (aneuploidies). Preimplantation genetic testing for aneuploidy (PGT-A) was therefore developed as an invasive method in order to select embryos for transfer, by biopsy and testing of the polar body or one or a few cells of the embryo.

Based on current evidence showing a lack of improvement in live birth rates or a decrease in miscarriages, routine use of PGT-A is not recommended. However, PGT-A may decrease time to pregnancy in specific patient groups.

Traffic light classification: yellow ●

#### *Timelapse imaging technology* (Chen *et al*., 2017)

As a result of the constant search for improvements in embryonic culture, the time-lapse imaging (TLI) system, which allows a non-invasive continuous assessment of embryonic morphokinetic parameters in a closed culture system, was developed. The TLI system promises to improve embryonic development by reducing oscillations in pH, humidity and temperature of the culture medium.

Traffic light classification: yellow ●

#### *Endometrial microbiome metagenomic analysis (EMMA)* (Clain & Devine, 2023)

The EMMA test, using next generation sequencing technology (NGS), analyzes the complete profile of the endometrial microbial environment, providing information on cultivable and non-culturable bacteria, thus favoring the clinical diagnosis.

Traffic light classification: red ●

#### *Analysis of chronic infectious endometritis (ALICE)* (Clain & Devine, 2023)

The ALICE exam makes the diagnosis of the bacteria present in the endometrial microbiome that cause chronic endometritis, as well as guides in the appropriate treatment.

Traffic light classification: red ●

#### *PAI-1 4G/5G polymorphism* (Chen *et al*., 2015)

The 4G/5G polymorphism of PAI-1 (plasminogen activator 1 inhibitor), a protein related to the blood clotting process, is a genetic alteration that may be associated with implantation failure, and pregnancy loss and complications.

Traffic light classification: red ●

#### Seminal plasma and platelet-rich plasma *(PRP) (Maleki-Hajiagha et al., 2020)*

During implantation, a state of maternal immune tolerance is required to prevent an immune attack on the developing embryo. Seminal plasma contains several proteins that interact with cervical and uterine epithelial cells, inducing active immune tolerance. Application of seminal plasma into the uterine cavity may improve pregnancy outcomes in women undergoing IVF. PRP is defined as the liquid part of the blood (plasma) containing many platelets that actively act in the clotting process. The blood supply to the endometrium and its thickness can be increased by intrauterine application of PRP.

Traffic light classification: red ●

#### *Screening hysteroscopy* (Kamath *et al*., 2019a)

Hysteroscopy is a minimally invasive surgical procedure that allows visualization and surgery of the uterine cavity. The benefit of hysteroscopy may extend beyond the treatment of intrauterine abnormalities. Irrigation of the cavity with saline may improve implantation and pregnancy rates, as saline mechanically washes away harmful molecules on the endometrial surface involved in its receptivity.

Although there is no high-quality evidence demonstrating the benefit of performing routine screening hysteroscopy in women undergoing IVF, a select group of women who have had one or more unsuccessful IVF attempts and a history of difficult embryo transfers may benefit (Kamath *et al*., 2019b). In patients with recurrent implantation failure, hysteroscopy may be beneficial, as shown in the meta-analysis by Cao *et al*. (2018).

Traffic light classification: red ●

#### *Growth hormone (GH)* (Sood *et al*., 2021)

Growth hormone is mainly secreted by the pituitary gland and participates in cell growth, development and metabolism, and its receptors are expressed in ovaries, oocytes, mammary glands, placenta, and uterus. GH increases the sensitivity of the ovaries to follicle-stimulating hormone and improves follicular development. GH has been applied in the treatment of infertility, especially for poor responders to ovarian stimulation.

Traffic light classification: yellow ●

#### *Aspirin* (Siristatidis *et al*., 2011)

The role of aspirin in women with infertility is controversial and the evidence is inconsistent. Proposed benefits of aspirin include improved uterine and ovarian blood flow, and prevention of thrombosis in the placental vasculature. It may also be a potent stimulator of interleukin-3 (IL-3) at low doses through its ability to increase the production of leukotrienes (proteins associated with successful pregnancy).

Traffic light classification: red ●

#### Heparin (Yang et al., 2018)

Low molecular weight heparin (LMWH) is a therapeutic intervention that has been suggested to improve the success of IVF/ICSI by modulating the physiological processes necessary for embryonic implantation (trophoblastic adhesion and invasion) and favoring placental angiogenesis during the first and second trimesters of pregnancy. Previous favorable effects of LMWH in women with thrombophilia precipitated the use of this drug in those without thrombophilia.

Traffic light classification: red ●

#### *Male antioxidants* (Showell *et al*., 2011)

Reactive oxygen species (ROS) are necessary for sperm function, however, an imbalance between their production and physiological antioxidant capacity can result in oxidative stress, which is deleterious to sperm quality. An extra dietary intake of antioxidants has been shown to be strongly associated with semen quality, which in turn could improve ART outcomes.

Traffic light classification: yellow ●

#### *Intravenous immunoglobulin* (Li *et al*., 2013)

During pregnancy, systemic immunological factors decrease the immunogenicity of the embryo and/or alter the maternal immune response to promote implantation and maintenance of early pregnancy. Several studies suggest that immunological factors may be involved in unexplained ART failures. Intravenous immunoglobulin is a monomeric preparation of IgG found in human blood, with anti-infective, anti-inflammatory and immunoregulatory properties, which has long been used for the treatment of immunodeficiency disorders. Several mechanisms have been proposed to improve implantation and maintenance of an early pregnancy by correcting the abnormal Th1/Th2 ratio (helper T cells), increasing antibody production, and inhibiting natural killer cell activity.

Traffic light classification: red ●

#### *Glucocorticoids* (Kalampokas *et al.,* 2017)

Adjunctive treatments during ovarian stimulation have been used to improve the patient’s response to stimulation and the outcome of ART. Glucocorticoids are a class of steroid hormones that have been reported to improve ovarian response and ART outcomes by suppressing androgen levels and altering cytokine levels, which may determine the ovarian response to controlled ovarian stimulation (COS).

Traffic light classification: red ●

### Motivation behind offering add-ons

Many investments are involved during the treatment of ART, including the physical and emotional ones, in addition to the financial ones in the case of Brazil where treatments are not funded by the health system nor reimbursed by health plans. Unfortunately, ART treatment fails more than it succeeds in getting the dreamed baby home. However, self-advertisement of fertility clinics emphasizing superior results and suggesting the realization of the dream of motherhood is not uncommon (Wilkinson *et al*., 2017; Hammarberg *et al*., 2018; Spencer *et al*., 2016). For this, many of them offer add-ons, either because of the genuine desire to increase the chance of pregnancy, or because of other motivations such as competition between clinics, patient retention, self-promotion, and extra gain, which are associated with the distortion of medical practice.

The truth is that there would be no ethical questions if add-ons were effective. None of these treatments were given the green light in a recent literature review carried out by the HFEA in the UK. Which brings us to certain ethical questions: Under what circumstances, if any, would it be acceptable to offer and sell complementary therapies that have questionable effectiveness and safety? How to regulate and inspect the use of these therapies?

### Current regulations for the use of add-ons

Current regulations for the use of add-ons are minimal and, in the case of Brazil and many other countries, non-existent. Self-regulation, together with market pressure, seems to be the standard for regulating innovations related to IVF treatment worldwide. Consequently, in countries where IVF treatment is carried out in state hospitals (Netherlands, Belgium, Slovenia, etc.) (ESHRE, 2017), the use of add-ons is believed to be lower. The HFEA has limited power to prevent the sale and pricing of add-ons in the UK (HFEA, 2016), as long as it considers the treatment dangerous in terms of safety, regardless of effectiveness. A consensus statement from the same authority advises that complementary treatments can be offered to patients even if evidence of efficacy is limited, provided they are properly informed (HFEA, 2019b). In case of total lack of information on efficacy and safety, the document advises which treatments can be offered to patients participating in research projects. In Australia, the Victorian Assisted Reproductive Treatment Authority, as well as the HFEA, advise patients that complementary treatments may not increase the chance of success of their IVF treatments. For the remaining nations, so far, there are no regulatory bodies acting on the dilemma of using these therapies.

### How to regulate add-ons and the role of informed consent

Recently, an ESHRE Journal Club discussion defined the term add-ons as “…procedures, techniques, or medications that may be used in addition to standard IVF protocols, generally in an attempt to increase success rates.” Journal Club participants further highlighted that add-ons should be labeled as procedures at additional cost, and as ‘experimental’ in the absence of evidence supporting their use, and that evidence for their use should be presented to patients (Wilkinson *et al*., 2019).

In the absence of regulations for the introduction of new procedures in the routine of ART and considering the propagation of these procedures in the media of fertility clinics themselves, the easiest and fastest way to contain the unbridled adoption of treatments complementary to IVF would be inform patients so that they can make a genuinely well-informed and autonomous decision about treatment. The present consensus agreed that the ideal scenario would be one in which all treatments offered to patients had been previously proven by robust scientific studies. However, in the management of exceptional situations, or in the face of several failures in the treatment of IVF, a treatment without scientific proof due to lack of data or evidence of high quality, or any other reasons, can be considered ethically acceptable and used (i) if based on a genuine desire to increase pregnancy rates, (ii) provided that the add-on in question does not cause proven harm to patients, (iii) after discussing with patients its advantages and disadvantages, quality or lack of scientific evidence, and the eventual costs involved (iv) with consent and consent of the patients, and (v) with due documentation.

The present consensus addressed another important aspect, which is the patient’s involvement in making informed decisions. The specialist physician has a greater contribution to the decision to use add-ons than the patient herself and must establish an acceptable limit for the use of supplements, not adopting experimental procedures with proven harm. The medical act is a medical responsibility, and therefore the doctor must determine the conduct and demolish idealizations that do not benefit the patient, justifying the points involved, in accordance with good clinical practice.

Regarding the propagation of add-ons on social networks, it was agreed that considering the positive and negative aspects, social networks are useful for patients as a “call to debate” about these techniques, and it is up to the specialist doctor to discuss with the patient on the treatment in question based on reliable information sources such as FEBRASGO, SPMR, SBRA, SBRH, RedLara, etc. The exposure of add-ons on social media can be considered ethical despite the lack of scientific evidence or consensus on their effectiveness, if the level of scientific evidence is exposed and clarified, the fact that they may be beneficial only in some specific cases, and the increase in the value of the treatment. However, there are still no adequate filters to moderate the information propagated by social networks, which can result in anxiety and other negative feelings in the patient.

## CONCLUSION

Most of these IVF adjuncts are being rushed into routine clinical practice with no clear evidence of benefit in most cases. It is important to make it clear that many of these add-ons are still evolving and that the traffic light colors may change with the emergence of new high-quality evidence. However, there are those who say that the absence of high-quality evidence over many years can be a sign that the treatment is not effective. Randomized clinical trials followed by well-designed meta-analyses should be conducted to assess the efficacy and safety of IVF supplements, as there is a paucity of information when considering the outcome of live births in clinical trials. Furthermore, the development of an evidence-driven system for introducing new treatments into the fertility clinic routine is imperative.

### Future perspectives

Establishing consensus-based treatment classifications may be an option. For example, a scoring tool was developed by the special interest groups ESHRE Ethics and Law, and Safety and Quality in Assisted Reproductive Technology to differentiate experimental, innovative and established treatments (Provoost *et al*., 2014), incorporating four domains that should have a threshold exceeded for a higher rating to be obtained: efficacy, safety, reliability and transparency of the procedure, and effectiveness.

To help patients make more informed decisions, we encourage them to ask themselves the five questions recommended by the Choose Wisely campaign (www.choosingwisely.org.au) before adopting an IVF supplement: “Do I really need this trial and treatment? or procedure?”, “What are the risks?”, “Are there simpler and safer options?”, “What happens if I do nothing” and “What are the costs?”.

It should also be thought how this information about complementary treatments in ART can reach patients. While it is expected that patients obtain all information from the fertility center where it is attended, the commercial environment can make impartiality a challenge, and it has been reported that in settings where IVF treatment is privately funded, self-regulation cannot be relied upon to protect patients from ineffective and unnecessary treatments (Hodson & Bewley, 2019).

For now, the best way to inform patients is to translate medical knowledge about supplements in a way that does justice to any risks and uncertainties.

## References

[r1] Ali SB, Jeelall Y, Pennell CE, Hart R, McLean-Tooke A, Lucas M. (2018). The role of immunological testing and intervention in
reproductive medicine: A fertile collaboration?. Am J Reprod Immunol.

[r2] ANVISA - Agencia Nacional de Vigilância
Sanitária (2022). 13° Relatório do Sistema Nacional de Procução de
Embriões - SisEmbrio.

[r3] Australian Health Practitioner Regulation Agency (2020). Social Media: How to Meet Your Obligations under the National
Law.

[r4] Bazarova NN, Choi YH. (2014). Self-Disclosure in Social Media: Extending the Functional
Approach to Disclosure Motivations and Characteristics on Social Network
Sites. J Commun.

[r5] Cain J, Romanelli F. (2009). E-professionalism: a new paradigm for a digital
age. Curr Pharm Teach Learn.

[r6] Cao H, You D, Yuan M, Xi M. (2018). Hysteroscopy after repeated implantation failure of assisted
reproductive technology: A meta-analysis. J Obstet Gynaecol Res.

[r7] Chen H, Nie S, Lu M. (2015). Association between plasminogen activator inhibitor-1 gene
polymorphisms and recurrent pregnancy loss: a systematic review and
meta-analysis. Am J Reprod Immunol.

[r8] Chen M, Wei S, Hu J, Yuan J, Liu F. (2017). Does time-lapse imaging have favorable results for embryo
incubation and selection compared with conventional methods in clinical in
vitro fertilization? A meta-analysis and systematic review of randomized
controlled trials. PLoS One.

[r9] Cheston CC, Flickinger TE, Chisolm MS. (2013). Social media use in medical education: a systematic
review. Acad Med.

[r10] Chretien KC, Goldman EF, Beckman L, Kind T. (2010). It’s your own risk: medical students’ perspectives on online
professionalism. Acad Med.

[r11] Clain E, Devine K. (2023). Endometrial receptivity, to test or not to test: the evidence on
contemporary assays. F S Rev.

[r12] Cornelisse S, Zagers M, Kostova E, Fleischer K, van Wely M, Mastenbroek S. (2020). Preimplantation genetic testing for aneuploidies (abnormal number
of chromosomes) in in vitro fertilisation. Cochrane Database Syst Rev.

[r13] Dai Y, Liu J, Yuan E, Li Y, Shi Y, Zhang L. (2022). Relationship Among Traditional Semen Parameters, Sperm DNA
Fragmentation, and Unexplained Recurrent Miscarriage: A Systematic Review
and Meta-Analysis. Front Endocrinol (Lausanne).

[r14] Datta AK, Campbell S, Deval B, Nargund G. (2015). Add-ons in IVF programme - Hype or Hope?. Facts Views Vis Obgyn.

[r15] ESHRE - European Society for Hum Reprod and Embryology (2017). The funding of IVF treatment.

[r16] Facione PA. (1990). Millbrae.

[r17] Gettig JP, Noronha S, Graneto J, Obucina L, Christensen KJ, Fjortoft NF. (2016). Examining Health Care Students’ Attitudes toward
E-Professionalism. Am J Pharm Educ.

[r18] Gholami-Kordkheili F, Wild V, Strech D. (2013). The impact of social media on medical professionalism: a
systematic qualitative review of challenges and
opportunities. J Med Internet Res.

[r19] Greysen SR, Chretien KC, Kind T, Young A, Gross CP. (2012). Physician violations of online professionalism and disciplinary
actions: a national survey of state medical boards. JAMA.

[r20] Grisham T. (2009). The Delphi technique: a method for testing complex and
multifaceted topics. Int J Manag Proj Bus.

[r21] Hall M, Hanna LA, Huey G. (2013). Use and views on social networking sites of pharmacy students in
the United Kingdom. Am J Pharm Educ.

[r22] Hammarberg K, Prentice T, Purcell I, Johnson L. (2018). Quality of information about success rates provided on assisted
reproductive technology clinic websites in Australia and New
Zealand. Aust N Z J Obstet Gynaecol.

[r23] HFEA - Human Fertilisation and Embryology Authority (2016). HFEA statement on fertility treatment “add-ons.”.

[r24] HFEA - Human Fertilization and Embryo Authority (2019a). Treatment add-ons with limited evidence.

[r25] HFEA - Human Fertilisation and Embryology Authority (2019b). The responsible use of treatment add-ons in fertility services: a
consensus statement.

[r26] Hodson N, Bewley S. (2019). Abuse in assisted reproductive technology: A systematic
qualitative review and typology. Eur J Obstet Gynecol Reprod Biol.

[r27] Hozyen M, Hasanen E, Elqusi K, ElTanbouly S, Gamal S, Hussin AG, AlKhader H, Zaki H. (2022). Reproductive Outcomes of Different Sperm Selection Techniques for
ICSI Patients with Abnormal Sperm DNA Fragmentation: a Randomized Controlled
Trial. Reprod Sci.

[r28] Kaczmarczyk JM, Chuang A, Dugoff L, Abbott JF, Cullimore AJ, Dalrymple J, Davis KR, Hueppchen NA, Katz NT, Nuthalapaty FS, Pradhan A, Wolf A, Casey PM. (2013). e-Professionalism: a new frontier in medical
education. Teach Learn Med.

[r29] Kalampokas T, Pandian Z, Keay SD, Bhattacharya S. (2017). Glucocorticoid supplementation during ovarian stimulation for IVF
or ICSI. Cochrane Database Syst Rev.

[r30] Kamath MS, Bosteels J, D’Hooghe TM, Seshadri S, Weyers S, Mol BWJ, Broekmans FJ, Sunkara SK. (2019a). Screening hysteroscopy in subfertile women and women undergoing
assisted reproduction. Cochrane Database Syst Rev.

[r31] Kamath MS, Mascarenhas M, Franik S, Liu E, Sunkara SK. (2019b). Clinical adjuncts in in vitro fertilization: a growing
list. Fertil Steril.

[r32] Lacey L, Hassan S, Franik S, Seif MW, Akhtar MA. (2021). Assisted hatching on assisted conception (in vitro fertilisation
(IVF) and intracytoplasmic sperm injection (ICSI)). Cochrane Database Syst Rev.

[r33] Lefebvre C, Mesner J, Stopyra J, O’Neill J, Husain I, Geer C, Gerancher K, Atkinson H, Harper E, Huang W, Cline DM. (2016). Social Media in Professional Medicine: New Resident Perceptions
and Practices. J Med Internet Res.

[r34] Lele C. (2016). The 3E Principle & Outsourcing.

[r35] Lensen S, Chen S, Goodman L, Rombauts L, Farquhar C, Hammarberg K. (2021a). IVF add-ons in Australia and New Zealand: A systematic assessment
of IVF clinic websites. Aust N Z J Obstet Gynaecol.

[r36] Lensen SF, Armstrong S, Gibreel A, Nastri CO, Raine-Fenning N, Martins WP. (2021b). Endometrial injury in women undergoing in vitro fertilisation
(IVF). Cochrane Database Syst Rev.

[r37] Li J, Chen Y, Liu C, Hu Y, Li L. (2013). Intravenous immunoglobulin treatment for repeated IVF/ICSI
failure and unexplained infertility: a systematic review and a
meta-analysis. Am J Reprod Immunol.

[r38] Linstone HA, Turoff M (2002). The Delphi Method: Techniques and Applications.

[r39] Maleki-Hajiagha A, Razavi M, Rouholamin S, Rezaeinejad M, Maroufizadeh S, Sepidarkish M. (2020). Intrauterine infusion of autologous platelet-rich plasma in women
undergoing assisted reproduction: A systematic review and
meta-analysis. J Reprod Immunol.

[r40] Mansfield SJ, Morrison SG, Stephens HO, Bonning MA, Wang SH, Withers AH, Olver RC, Perry AW. (2011). Social media and the medical profession. Med J Aust.

[r41] McDowell S, Kroon B, Ford E, Hook Y, Glujovsky D, Yazdani A. (2014). Advanced sperm selection techniques for assisted
reproduction. Cochrane Database Syst Rev.

[r42] Monitor Mercantil (2021). Brasil é o terceiro país que mais usa redes sociais no
mundo.

[r43] Moorhead SA, Hazlett DE, Harrison L, Carroll JK, Irwin A, Hoving C. (2013). A new dimension of health care: systematic review of the uses,
benefits, and limitations of social media for health
communication. J Med Internet Res.

[r44] Moretti FA, Oliveira VE, Silva EM. (2012). Access to health information on the internet: a public health
issue?. Rev Assoc Med Bras (1992).

[r45] Murugesu S, Saso S, Jones BP, Bracewell-Milnes T, Athanasiou T, Mania A, Serhal P, Ben-Nagi J. (2017). Does the use of calcium ionophore during artificial oocyte
activation demonstrate an effect on pregnancy rate? A
meta-analysis. Fertil Steril.

[r46] National Council of State Boards of Nursing (2020). Social Media Guidelines for Nurses.

[r47] Nazareth RT, de Almeida JJG, Bastos AT. (2020). Utilização do whatsapp e o parecer CFM nº
14/2017. Rev Univ Ibirapuera.

[r48] Neville P, Waylen A. (2015). Social media and dentistry: some reflections on
e-professionalism. Br Dent J.

[r49] O’Connor S. (2017). Using social media to engage nurses in health policy
development. J Nurs Manag.

[r50] O’Connor S, Jolliffe S, Stanmore E, Renwick L, Booth R. (2018). Social media in nursing and midwifery education: A mixed study
systematic review. J Adv Nurs.

[r51] Osborne J, Collins S, Ratcliffe M, Millar R, Duschl R. (2003). What “ideas-about-science” should be taught in school science? A
Delphi study of the expert community. J Res Sci Teach.

[r52] Provoost V, Tilleman K, D’Angelo A, De Sutter P, de Wert G, Nelen W, Pennings G, Shenfield F, Dondorp W. (2014). Beyond the dichotomy: a tool for distinguishing between
experimental, innovative and established treatment. Hum Reprod.

[r53] Rimmer A. (2017). Doctors’ use of Facebook, Twitter, and WhatsApp is the focus of
28 GMC investigations. BMJ.

[r54] Setton MGJ. (2002). Family, school, and media: a field with new
configurations. Educ Pesqui.

[r55] Showell MG, Brown J, Yazdani A, Stankiewicz MT, Hart RJ. (2011). Antioxidants for male subfertility. Cochrane Database Syst Rev.

[r56] Siristatidis CS, Dodd SR, Drakeley AJ. (2011). Aspirin for in vitro fertilisation. Cochrane Database Syst Rev.

[r57] Sood A, Mohiyiddeen G, Ahmad G, Fitzgerald C, Watson A, Mohiyiddeen L. (2021). Growth hormone for in vitro fertilisation (IVF). Cochrane Database Syst Rev.

[r58] Sosa JA. (2020). Editorial: Doubling Down on Diversity in the Wake of the
#MedBikini Controversy. World J Surg.

[r59] Spencer EA, Mahtani KR, Goldacre B, Heneghan C. (2016). Claims for fertility interventions: a systematic assessment of
statements on UK fertility centre websites. BMJ Open.

[r60] Sterling M, Leung P, Wright D, Bishop TF. (2017). The Use of Social Media in Graduate Medical Education: A
Systematic Review. Acad Med.

[r61] Teixeira DM, Hadyme Miyague A, Barbosa MA, Navarro PA, Raine-Fenning N, Nastri CO, Martins WP. (2020). Regular (ICSI) versus ultra-high magnification (IMSI) sperm
selection for assisted reproduction. Cochrane Database Syst Rev.

[r62] Mitscherlich A, Mielke F., The Nuremberg Code (1947). Doctors of infamy: the story of the Nazi medical crimes.

[r63] Toral N. (2006). Stages of change and its relationship with dietary intake among
adolescents.

[r64] Vaiarelli A, Cimadomo D, Petriglia C, Conforti A, Alviggi C, Ubaldi N, Ledda S, Ferrero S, Rienzi L, Ubaldi FM. (2020). DuoStim - a reproducible strategy to obtain more oocytes and
competent embryos in a short time-frame aimed at fertility preservation and
IVF purposes. A systematic review. Ups J Med Sci.

[r65] Wechsler R, Anção MS, de Campos CJ, Sigulem D. (2003). [Computing in medical practice]. J Pediatr (Rio J).

[r66] Wilkinson J, Vail A, Roberts SA. (2017). Direct-to-consumer advertising of success rates for medically
assisted reproduction: a review of national clinic websites. BMJ Open.

[r67] Wilkinson J, Malpas P, Hammarberg K, Mahoney Tsigdinos P, Lensen S, Jackson E, Harper J, Mol BW. (2019). Do à la carte menus serve infertility patients? The ethics
and regulation of in vitro fertility add-ons. Fertil Steril.

[r68] World Medical Association (2001). World Medical Association Declaration of Helsinki. Ethical
principles for medical research involving human subjects. Bull World Health Organ.

[r69] Yang XL, Chen F, Yang XY, Du GH, Xu Y. (2018). Efficacy of low-molecular-weight heparin on the outcomes of in
vitro fertilization/intracytoplasmic sperm injection pregnancy in
non-thrombophilic women: a meta-analysis. Acta Obstet Gynecol Scand.

[r70] Zaat T, Zagers M, Mol F, Goddijn M, van Wely M, Mastenbroek S. (2021). Fresh versus frozen embryo transfers in assisted
reproduction. Cochrane Database Syst Rev.

